# NonLinear Effects of Environmental Regulation on Eco-Efficiency under the Constraint of Land Use Carbon Emissions: Evidence Based on a Bootstrapping Approach and Panel Threshold Model

**DOI:** 10.3390/ijerph16101679

**Published:** 2019-05-14

**Authors:** Haoran Yang, Hao Zheng, Hongguang Liu, Qun Wu

**Affiliations:** College of Public Administration, Nanjing Agricultural University, Nanjing 250030, China; yanghr90@ou.edu (H.Y.); 2015209025@njau.edu.cn (H.Z.); liuhg@njau.edu.cn (H.L.)

**Keywords:** environmental regulation, eco-efficiency, carbon emission, bootstrapping approach, panel threshold model

## Abstract

Eco-efficiency has been receiving attention worldwide, and the effective implementation of environmental regulations (ERs) has become crucial to regional eco-efficiency. This paper uses a method combining mixed directional distance function and bootstrapping approach to investigate the spatial and temporal distribution characteristics of eco-efficiency under the constraint of land use carbon emission in China from 2004 to 2016. The nonlinear relationship between ER and eco-efficiency is observed with a panel threshold model. Results from empirical tests reveal that eco-efficiency in China during the study period has an upward trend, and the spatial and temporal distribution of eco-efficiency is unbalanced and concentrated. Technical innovation and land marketization (LM) shows double threshold, whereas industrial structure (IS) has a single threshold effect. LM has a promotional effect on eco-efficiency, which differs in the promotion before and after promotion across the threshold value. Reasonable ER can reduce cost by stimulating the innovation of green production technology and achieves a win-win situation between environment and output. This finding further verifies that the ER for eco-efficiency under the constraint of land use carbon emission conforms to the Porter hypothesis. The effect of ER on eco-efficiency changes from negative to positive with the increase of IS level. Adjusting the ownership structure and increasing the proportion of green achievements in the promotion and assessment of officials are important measures in the upgrading of eco-efficiency.

## 1. Introduction

The definition of eco-efficiency was first proposed by Schaltegger and Sturm in 1990; the World Business Council for Sustainable Development (WBCSD) defines it as the product of price competition advantage, which can satisfy human needs and improve living standards, as well as the impact on the ecological environment and resource consumption gradually reduced to and forecast of earth bearing level of the same level and achieve the coordinated development of environment and society [1]. International organizations have been committed to carbon emission reduction for the mitigation of global warming [2,3]. In China, energy saving and emission reduction have attracted considerable interest in view of environmental pollution and excessive resource consumption due to rapid economic growth and urban expansion. The focus of CO_2_ emission reduction is on fossil energy consumption, whereas CO_2_ generated by land use changes caused by human activities is ignored [4,5]. Greenhouse gas emissions from human activities are the main cause of global warming [6], CO_2_ emissions accounted for 78% during the period from 1970 to 2010 [7]. Governments are responsible for identify instruments that can ameliorate global warming. According to statistics, China became the world’s largest emitter of CO_2_ in 2007 and promised to reach the peak of carbon emission around 2030 and reduce emission intensity by 60–65% relative to that in 2005 [8,9]. Environmental regulations (ERs) are expected to play an increasingly essential role in achieving carbon emission reduction targets and meet the requirement of resource saving and environment protection.

ERs are classified into three types, namely, command-and-control (C & C), market-based, and reluctant regulations respectively. Under C & C regulations, government determines the allocation of pollutant quotas. Over-polluting enterprises are penalized under compulsive regulations. To ensure that enterprises comply with ERs, China enacted relevant environmental laws and regulations, as well as administrative commands. China’s government has implemented stringent market-based ERs, such as pollution control subsidy, waste water discharge fees, and excess discharge fees, since its entry into the World Trade Organization (WTO). C & C regulations is remain the most popular ER in regulating the environmental problems in China despite its ineffectiveness in improving country’s ecological environment after its accession to the WTO [10]. Environment regulation on environment protection has two conductive paths: industrial structure (IS) and official competition (COM). It analyzes the restrictive factors of industrial expansion from the indirect function path increases the cost of operating energy-intensive industries and thus promotes the upgrading of IS, and reduces air pollution.

Previous studies have explored the impact of ERs on eco-efficiency based on a linear regression model, but the results have limited practicality [11,12,13,14] and few studies have focused on the nonlinear connection between ER and eco-efficiency. Considering the research gap, this paper is aimed at the following aspects: First, carbon emission caused by land use activities is taken into the indicator system for estimating of the eco-efficiency under the constraint of land use carbon emission. Second, the indirect effect of ER is explored by using a panel threshold model. Third, IS, research and development (R & D) and land marketization (LM) are used as threshold variables. This paper investigates regional differences and proposes targeted policies based on the characteristics of regional eco-efficiency.

The paper is organized as follows: Section 2 provides an overview of ER and eco-efficiency. Section 3 describes the eco-efficiency estimation method and panel threshold regression. model Section 4 outlines the results and analysis of eco-efficiency and the threshold regression. Section 5 concludes the work.

## 2. Literature Review

The concept of eco-efficiency was defined as the ratio of outputs divided by the Organization for Economic Co-operation and Development (OECD) [15]; the value of products and services produced by the enterprises represent the outputs, whereas the sum of environmental pressures generated by the enterprises represent the inputs. Previous research on eco-efficiency mainly estimate the eco-efficiency of industries [16], enterprises [17,18], and different regions [19,20]. To date, the influencing factors of eco-efficiency have focused on science and technology investment, foreign direct investment (FDI), IS, and urbanization [21]. Environmental regulation is also an important factor in accelerating the technical innovation (RD) and promoting the environment and economic level of enterprises [22,23,24], but studies have rarely focused on ER and eco-efficiency interaction. Furthermore, the degree of ER and the value of eco-efficiency vary by region. Thus, studying the mechanism of how ER affects eco-efficiency is significant in different regions in China as well as helpful for environmental enhancement.

In addition to the studies, the effects of ERs on environmental pollution has also been studied in three aspects. The first one is the “cost compliance theory,” which means that raising the level of ER encroaches on parts of production costs and reduces economic outputs, thereby affecting economic aggregate. Laplante and Rilstone [25] researched the pulp and paper product industry in Canada to verify the negative relationship between pollution emission and ER. Dasgupta et al. studied the relationship between ER and pollutant discharge, results showed that regulations could decrease the pollutant discharge effectively through changing the emission level [26]. Compared with the level of pollutant emission, environmental management should be more strictly strengthened for pollutant control. Jaffe and Stavins [27] concluded that ER increases the costs on enterprises and restricted the capital to improve the potential for technological innovation. Levinson and Taylor [28] found that the production cost of enterprises under the constraints of ERs increases due to the increase in pollution control costs and the price of production factors and reduces the innovation capability and competitiveness of enterprises and ultimately adversely affects enterprise and industrial performance. Xie et al. found that current C & C and market-oriented ERs can significantly promote regional green productivity [29]. The second one aspect “Porter hypothesis,” that is, reasonable ER can stimulate the innovation compensation effect of enterprises, which not only can offset the loss of compliance cost, but also can produce technology diffusion and structure upgrade effect. Nadeau concluded that environmental regulations could cut down the time spending in violating factory standards [30]. Daron et al. found that dynamic ERs can promote technological advances in low-emission machinery [31]. Therefore, increasing the intensity of ER would promote the demand of clean technology for polluting enterprises. Moreover, reducing the demand for pollution-intensive technologies would contribute to establishing the direction of technological innovation in the next stage. The last one is uncertainty theory. Due to the heterogeneity of ER quality and industry characters, the functional relations among them is uncertain. Lanoie et al. empirically studied the impact of environmental regulation on the productivity of 17 manufacturing industries in Quebec, Canada [32]. The results showed that the effect of ER on the productivity of manufacturing industries was positive in the long term and negative in the short term. Yuan and Xie reported that a U-shaped relationship existed between cost-type regulation and green industrial productivity, whereas a negative linear relationship existed between investment-type regulation and green industrial productivity [33].

Most of the existing studies analyzed the relationship between ER, as well as economic growth and pollutant emission, but the research on the influence of LM (the ratio of the number of land lots to the total number of transferred land lots in the primary land market) and IS adjustment on pollutant emission was rarely involved. The previous studies on the relationship among the three still have the following shortcomings: First, carbon emission constraints were not included in the analysis framework of eco-efficiency estimation, which is an important factor affecting the ecological environment. Second, the efficiency value calculated based on Data Envelopment Analysis (DEA) method led to the deviation of the actual efficiency level. Therefore, based on the shortcomings of existing studies, this paper uses the provincial panel data from 2004 to 2016 and bootstrap method to modify the value of efficiency, and conducts an empirical analysis on the threshold effect of the openness to the outside world and IS adjustment on eco-efficiency under the background of ER.

## 3. Methodology and Data Specification

### 3.1. Eco-Efficiency Estimation Method

#### 3.1.1. Mixed Directional Distance Function

Directional distance function can solve the problems of unsatisfactory output efficiency evaluation, which is widely used in considering the purpose of the output efficiency evaluation [34,35,36]. It can be used to calculate the optimal solution of the production feasibility set to reflect the use of environmental technology in the process of economic activities after determining the investment portfolio. Directional distance is expressed as follows:

(1) $$\vec{D_{0}}\left(x,y,b;g\right)=max\left\{\beta ∶\left(y,b\right)+\beta g\epsilon P\left(x\right)\right\}$$

where *g* = (*y*, −*b*) is the direction vector of output horizontal expansion, and β is the value of the directional distance function. The maximum value of the desirable output (*y*) and the minimum value of the undesirable output (*b*) are obtained by taking the set direction vector as the weight. If the radial and output angle DEA are used to calculate the directional distance function, then given nonzero relaxation between input and output, the efficiency measurement value will be higher than the actual efficiency of the evaluation object. When the output angle DEA measurement efficiency cannot reflect a certain output, the input can be reduced. The non-radial and non-angular SBM (Slacks-Based Measure) directional distance function is used to obtain the non-efficiency score by maximizing the average relaxation of all inputs and outputs. When the input or output changes in the same proportion, this method may be lower than the actual efficiency value of the evaluation object. [37]. Therefore, the mixed directional distance function proposed by Tone will be applied in this paper to avoid their defects [38].

Input matrix $X_{im}^{t}$ is decomposed into radial one $X_{im}^{Rt}\epsilon R_{+}^{l_{1}}$ and radial one $X_{im}^{NRt}\epsilon R_{+}^{l_{2}}\left(l_{1}+l_{2}=l\right)$, namely $X_{im}^{t}=\left(\begin{array}{c} X_{im}^{Rt}\\ X_{im}^{NRt} \end{array}\right)$. The desirable output matrix $Y_{i}^{t}$ is decomposed into radial one $Y_{i}^{Rt}\epsilon R_{+}^{s_{1}}$ and nonradial one $Y_{i}^{NRt}\epsilon R_{+}^{s_{2}}$(s1 + s2 = s), namely, $Y_{i}^{t}=\left(\begin{array}{c} Y_{i}^{Rt}\\ Y_{i}^{NRt} \end{array}\right)$. Similarly, the undesirable output matrix $E_{in}^{t}$ is divided into radial one $E_{in}^{Rt}\epsilon R_{+}^{h_{1}}$ and the nonradial part $E_{in}^{NRt}\epsilon R_{+}^{h_{2}}$(h1 + h2 = h), namely, $E_{in}^{t}=\left(\begin{array}{c} E_{in}^{Rt}\\ E_{in}^{NRt} \end{array}\right)$. The direction vector is decomposed into six vectors, $g=\left(g_{X_{im}^{t},}g_{X_{im}^{NRt},}g_{Y_{i}^{t},}g_{Y_{i}^{Nt},}g_{E_{in}^{t},}g_{E_{in}^{NRt},}\right)$, finally, extending to the mixed directional distance function. The form of linear programming is as follows:

$$\vec{D_{0}}\left(X_{im}^{t},Y_{i}^{t},E_{in}^{t};g\right)=\max\;w^{T}\cdot \beta$$

$$s.t\;X_{im}^{t}\lambda \leq X_{im}^{t}+\beta_{X_{im}^{Rt}}\cdot diag\left(g_{X_{im}^{Rt}}\right)$$

$$X_{im}^{NRt}\lambda \leq X_{im}^{NRt}+\beta_{X_{im}^{NRt}}\cdot diag\left(g_{X_{im}^{NRt}}\right)$$

$$Y_{i}^{Rt}\lambda \geq Y_{i}^{Rt}+\beta_{Y_{i}^{Rt}}\cdot diag\left(g_{Y_{i}^{Rt}}\right)$$

$$Y_{i}^{NRt}\lambda \geq Y_{i}^{NRt}+\beta_{Y_{i}^{NRt}}\cdot diag\left(g_{Y_{i}^{NRt}}\right)$$

$$E_{in}^{Rt}\lambda =E_{in}^{Rt}+\beta_{E_{in}^{Rt}}\cdot diag\left(g_{E_{in}^{Rt}}\right)$$

$$E_{in}^{NRt}\lambda =E_{in}^{NRt}+\beta_{E_{in}^{NRt}}\cdot diag\left(g_{E_{in}^{NRt}}\right)$$

$$\beta =\beta \cdot sgn{\left(\left|g\right|\right)}^{T},\;\geq 0,$$

where $w={\left(w_{X_{im}^{Rt},}w_{X_{im}^{NRt},}w_{Y_{i}^{Rt},}w_{Y_{i}^{NRt},}w_{E_{in}^{Rt},}w_{E_{in}^{NRt}}\right)}^{T}$ represents the standardized weight vector corresponding to radial input, non-radial input, radial desirable output, non-radial desirable output, radial undesirable output, and non-radial undesirable output; λ represents the weight of input X or output Y. $\beta ={\left(\beta_{X_{im}^{Rt},}\beta_{X_{im}^{NRt},}\beta_{Y_{i}^{Rt},}\beta_{Y_{i}^{NRt},}\beta_{E_{in}^{Rt},}\beta_{E_{in}^{NRt}}\right)}^{T}\geq 0$.

#### 3.1.2. Bootstrap–DEA Approach

The DEA evaluates the performance through estimating the true and unobservable production frontier based on finite sample, which may give rise to a corresponding efficiency metric which is very sensitive to the sampling variations of the obtained frontier. However, the traditional DEA method could not show the characteristics of non-parameter statistics well and the results of bootstrap–DEA model are more reliable and accurate. To solve this problem, a smooth bootstrap method proposed by Simar and Wilson was used to study the sampling characteristics of DEA estimators, and the robustness of DEA point estimators was evaluated by constructing confidence intervals [39]. The basic idea of the bootstrap method is to carry on the numerical stimulation by using the original samples and generate many stimulated samples to be used to be calculated the DEA efficiency values. The sample distribution obtained by the bootstrap method can be used for simulating the distribution of the original sample estimator, correct the deviation of the values of efficiency, and provide the confidence interval of the measured efficiency values to avoid the error of the efficiency evaluation by the DEA model and the problem of statistic test. Thus, this paper analyzes the eco-efficiency value after rectification and the estimation steps are as follows:

(1) Calculate the original efficiency scores ${\hat{\beta }}_{m,i}^{X-t},\;{\hat{\beta }}_{i}^{Y-t},\;{\hat{\beta }}_{n,i}^{E-t}\;\left(i=1,\ldots Z;\;m=1,\ldots M;\;n=1,\ldots N;\;t=1,\ldots T\right)$ by solving the linear programming model (1).

(2) Generate a random sample ${\hat{\beta }}_{m,1b}^{X-t}\ldots {\hat{\beta }}_{m,Zb}^{X-t};\;{\hat{\beta }}_{1b}^{Y-t}\ldots {\hat{\beta }}_{Zb}^{Y-t};\;{\hat{\beta }}_{n,1b}^{E-t}\ldots {\hat{\beta }}_{n,Zb}^{E-t}$ with replacement from ${\hat{\beta }}_{m,1}^{X-t}\ldots {\hat{\beta }}_{m,Z}^{X-t};\;{\hat{\beta }}_{1}^{Y-t}\ldots {\hat{\beta }}_{Z}^{Y-t};\;{\hat{\beta }}_{n,1}^{E-t}\ldots {\hat{\beta }}_{n,Z}^{E-t}$.

(3) Take desirable output (Y) as an example, and smooth the sampled values using the following formula:

(2) $${\tilde{\beta }}_{i}^{Y-t,*}=\left\{\begin{array}{c} \beta_{ib}^{Y-t}+h^{Y-t}\varepsilon_{i}^{Y-t,*}\;if\;\beta_{ib}^{Y-t}+h^{Y-t,*}\leq 1\\ 2-\beta_{ib}^{Y-t}-h^{Y-t}\varepsilon_{i}^{Y-t,*}\;otherwise \end{array}\right\}\;\varepsilon_{i}^{*}\sim N\left(0,1\right)$$

where $h^{Y-t}$ and $\varepsilon_{i}^{Y-t,*}$ represent a smoothing parameter and a randomly drawn error term respectively. For the estimation of $h^{Y-t}$, this study maximizes a likelihood cross-referencing function using methods developed by Simar and Wilson.

(4) Take desirable output (Y) as an example, and obtain the corrected smoothed bootstrap sample by adjusting the smoothed sampled values using the following formula:

(3) $$\beta_{i}^{Y-t,*}={\bar{\beta }}^{Y-t}+\frac{{\tilde{\beta }}_{i}^{Y-t,*}-{\bar{\beta }}_{i}^{Y-t}}{\sqrt{\frac{1+{\left(h^{Y-t}\right)}^{2}}{{\tilde{\sigma }}_{\beta^{Y-t}}^{2}}}}$$

where $\bar{\beta }[\bar{\beta }=\left(\frac{1}{n}\right)\sum_{i=1}^{n}\beta_{ib}^{Y-t}]$ denotes the average resampled value and ${\overset{˘}{\sigma }}_{\beta^{Y-t}}^{2}[{\overset{˘}{\sigma }}_{\beta^{Y-t}}^{2}=\left(\frac{1}{n}\right)\sum_{i=1}^{n}{\left({\hat{\beta }}_{ib}^{Y-t}-{\bar{\beta }}_{ib}^{Y-t}\right)}^{2}]$ is the variance estimate of the measured efficiencies ${\hat{\beta }}_{i}^{Y-t}$.

(5) Adjust the original desirable output using the ratio $R_{m,ib}^{t*}=\left[\frac{1-{\hat{\beta }}_{m,i}^{X-t}}{1-\beta_{m,i}^{X-t,*}}\right]X_{m,i}^{t},\;Y_{ib}^{t*}=\left[\frac{1+{\hat{\beta }}_{i}^{Y-t}}{1+\beta_{i}^{Y-t,*}}\right]Y_{i}^{t},\;E_{n,ib}^{t*}=\left[\frac{1-{\hat{\beta }}_{n,i}^{E-t}}{1-\beta_{n,i}^{E-t,*}}\right]E_{n,i}^{t}$.

(6) Calculate the bootstrapped efficiency ${\hat{\beta }}_{m,i}^{X-t,*}$, ${\hat{\beta }}_{i}^{Y-t,*}$, ${\hat{\beta }}_{n,i}^{E-t,*}$ by solving the DEA model (1) using the pseudo-variable inputs obtained in Step 5.

(7) Repeat Steps 2–6 B times to obtain robust efficiency scores ${\hat{\beta }}_{m,ib}^{X-t,*}$, ${\hat{\beta }}_{ib}^{Y-t,*}$, ${\hat{\beta }}_{n,ib}^{E-t,*}$(*i* = 1,…Z; *m* = 1,…M; *n* = 1,…N; *t* = 1,…T).

(8) Compute confidence intervals for the performance indicators. Calculate the bias of the original estimate using the following formula:

$$\tilde{bıas}\left({\hat{\beta }}_{m,i}^{X-t}\right)=B^{-1}\sum_{b=1}^{B}{\tilde{\beta }}_{m,ib}^{X-t,*}-{\hat{\beta }}_{m,i}^{X-t},$$

$$\tilde{bıas}\left({\hat{\beta }}_{i}^{Y-t}\right)=B^{-1}\sum_{b=1}^{B}{\tilde{\beta }}_{ib}^{Y-t,*}-{\hat{\beta }}_{i}^{Y-t},$$

$$\tilde{bıas}\left({\hat{\beta }}_{n,i}^{E-t}\right)=B^{-1}\sum_{b=1}^{B}{\tilde{\beta }}_{n,ib}^{E-t,*}-{\hat{\beta }}_{n,i}^{E-t}.$$

Calculate the bias corrected estimator of the true value of $\beta_{i}$ using the following formula:

$${\tilde{\beta }}_{m,i}^{X-t}={\hat{\beta }}_{m,i}^{X-t}-\tilde{bıas}\left({\hat{\beta }}_{m,i}^{X-t}\right)=2{\hat{\beta }}_{m,i}^{X-t}-B^{-1}\sum_{b=1}^{B}{\tilde{\beta }}_{m,ib}^{X-t,*},$$

$${\tilde{\beta }}_{i}^{Y-t}={\hat{\beta }}_{i}^{Y-t}-\tilde{bıas}\left({\hat{\beta }}_{i}^{Y-t}\right)=2{\hat{\beta }}_{i}^{Y-t}-B^{-1}\sum_{b=1}^{B}{\tilde{\beta }}_{ib}^{Y-t,*},$$

$${\tilde{\beta }}_{n,i}^{E-t}={\hat{\beta }}_{n,i}^{E-t}-\tilde{bıas}\left({\hat{\beta }}_{n,i}^{E-t}\right)=2{\hat{\beta }}_{n,i}^{E-t}-B^{-1}\sum_{b=1}^{B}{\tilde{\beta }}_{n,ib}^{E-t,*}.$$

Take desirable output (Y) as an example, and compute the confidence intervals as follows:

$$P_{r}=-{\tilde{b}}_{\alpha }\leq \beta_{ib}^{Y-t,*}-{\hat{\beta }}_{i}^{Y-t}\leq -{\tilde{a}}_{\alpha })=1-\alpha$$

$$P_{r}=-{\tilde{b}}_{\alpha }\leq {\hat{\beta }}_{i}^{Y-t}-\beta_{i}^{Y-t}\leq -{\tilde{a}}_{\alpha })\approx 1-\alpha$$

$${\hat{\beta }}_{i}^{Y-t}+{\tilde{a}}_{\alpha }\leq \beta_{i}^{Y-t}\leq {\hat{\beta }}_{i}^{Y-t}+{\tilde{b}}_{\alpha }$$

According to the above steps, we can obtain the corrected efficiency values and 95% confidence interval.

#### 3.1.3. Data Specification

China has 34 provincial administrative regions: four municipalities (Beijing, Tianjin, Shanghai, and Chongqing), five autonomous regions (Inner Mongolia, Guangxi, Tibet, Ningxia, and Xinjiang), 23 provinces (including Taiwan), and two special administrative regions (Hong Kong and Macau). China can be further divided into seven economy-geography areas: East (Shanghai, Jiangsu, Zhejiang, Anhui, Fujian, Jiangsu, and Shandong), North (Beijing, Tianjin, Shanxi, Hebei, and Inner Mongolia), Central (Hebei, Hubei, and Hunan), South (Guangdong, Guangxi, and Hainan), Northeast (Heilongjiang, Jilin, and Liaoning), Northwest (Shaanxi, Gansu, Ningxia, Qinghai, and Xinjiang), Southwest (Chongqing, Sichuan, Guizhou, Yunnan, and Tibet). Lacking of data from Tibet, Hong Kong, Macau, and Taiwan, 30 provincial regions remain to be analyzed. Available statistical data of China generally have a lag of 1–2 years, and the research period is from 2004 to 2016. To measure eco-efficiency comprehensively and accurately, the variables (Table 1) used in this paper include the following:

(1). Desirable Output

The output values ($Y_{it}$) are measured by real Goss Domestic Production (GDP) at the provincial level in the unit of 100 million renminbi (RMB) and are converted to the 2004 constant price using the GDP deflator. The data can be obtained from the China Statistical Yearbook (2005–2017).

(2). Undesirable Output

The types of land involved in this paper include cultivated land, construction land, woodland, garden land, grassland, water area and unused land. The carbon sink mainly comes from the net CO_2_ from the atmosphere into the ecosystem. Carbon source mainly comes from the carbon produced by energy consumption, industrial production, transportation and agricultural production. Since the perspective of this paper is based on the carbon emission caused by the change of land use type caused by human activities, the carbon emission of construction land only considers the carbon emission generated by energy consumption. Carbon source mainly comes from the carbon produced by energy consumption, industrial production, transportation and agricultural production, the carbon emission of this paper is generated by the energy consumption of construction land as the main carbon source. We refer to the methods of Intergovernmental Panel on Climate Change (IPCC, 2007) and Energy Research Institute of National Development and Reform Commission in China (2003) to estimate the CO_2_ emissions generated by construction land in various provinces through relevant calculation formulas [4,40]. The calculation methods are as follows:

(4) $$E_{g}=E_{f}+E_{m}+E_{i}=G_{f}\cdot A+\left(A_{m}\cdot B+W_{m}\cdot C\right)+A_{i}\cdot D$$

(5) $$E_{k}=\sum e_{i}=\sum T_{i\cdot }\delta_{i}$$

(6) $$E_{t}=\sum E_{ti}=\sum E_{ni\cdot }\cdot \theta_{i}\cdot f_{i}$$

(7) $$E=E_{g}+E_{k}+E_{t}$$

where $E_{g}$ represents the carbon emission of cultivated land; $E_{f}$ stands for carbon emissions from fertilizer use; $E_{m}$ represents the carbon emissions generated by the production and use of agricultural machinery; $E_{i}$ represents the carbon emission in the irrigation process; $E_{i}$ is the amount of fertilizer used; $A_{m}$ represents the total sown area of crops; $W_{m}$ represents the total power of agricultural machinery; $A_{i}$ is irrigation area; $E_{k}$ represents the total carbon absorption; $e_{i}$ represents the amount of carbon absorption generated by different land use types; $T_{i\cdot }$ represents the area of the functional land type of carbon sink; $\delta_{i}$ is the carbon emission (absorption) coefficient of different land types, with positive carbon emission and negative carbon absorption. $E_{t}$ represents the carbon emission of construction land; $E_{ti}$ represents various energy carbon emissions; $E_{ni\cdot }$ represents the consumption of various types of energy; $\theta_{i}$ represents the coefficients of various energy transformations to standard coal; $f_{i}$ represents the carbon emission coefficient of various energy sources. E represents the net land use carbon emissions.

Fossil fuels are consumed from raw coal, crude oil and natural gas, with carbon factors of 0.7476, 0.5854, and 0.4479 tC/tce, respectively. Cultivated land is carbon source and carbon sink. The production and use of agricultural irrigation, chemical fertilizers, agricultural film, and pesticides and the transportation of agricultural machinery in the production and management process produce much more carbon emissions than carbon absorption. The carbon emission conversion coefficients (A, B, C, D) in the process of fertilizer, seeding, usage of agricultural machinery, and irrigation are 895.6 kg/t, 16.47 kg/hm^2^, 0.18 kg/kw, and 266.48 kg/hm^2^, respectively [41]. This paper only considers the carbon source of cultivated land. According to the complex land use structure characteristics in China, the factors of carbon absorption of woodland, grassland, garden, water area, and unused land are −6.44, −0.39, −6.44, −0.245, and −0.05 t/hm^2^ a, respectively [42].

The calculated standard energy carbon emission is the sum of the total consumption of various types of energy and the product of energy conversion standard coal coefficient and energy carbon emission coefficient. The selection of coefficients is calculated with reference to the coefficient values in the IPCC report. The carbon emissions of cultivated land are calculated by the use of chemical fertilizer, total planting area of crops, total power of agricultural machinery and irrigation area multiplied by their conversion coefficients. The carbon absorption of woodland, grassland, garden, water area and unused land are calculated by the product of their area and corresponding coefficients. All data of energy terminal consumption and conversion are taken from the regional energy balance table (real quantity) in the China Energy Statistical Yearbook (2005–2016). The data of land use area in each province are obtained from China Land and Resources Statistical Yearbook (2004–2016).

(3). Factor Inputs

The data of capital investment for each province are denoted by the capital stock. The method proposed by Shan is more scientific in terms of depreciation rate and base period capital stock calculation method for improvement based on the method of Zhang [43,44]. In this paper, the depreciation rate is 10.96%, and the base period is calculated by the sum of the average annual growth rate and the depreciation rate of the total amount of real fixed capital formation in the five years after the base period. The related data are derived from historical data of China’s GDP 1952–2004, China Statistical Yearbook (2005–2016), and compilation of statistical data of the 60 years of new China.

The data on labor inputs for each province are denoted by the number of employees in three industries taken from the China Provincial Statistical Yearbooks (2005–2017) at yearend.

Energy consumption contributes to Chinese economic development. The data on energy inputs for each province are denoted by the total energy consumption characterization taken from the China Energy Yearbook Statistical Yearbooks (2005–2017). Due to the differences in regional economic development level and geographic characteristics, the determination of eco-efficiency index system in this paper follows the practice of You [45]. Finally, fixed capital stock is selected as capital input, total employment as labor input, and total energy consumption as energy input.

### 3.2. Threshold Regression Model

#### 3.2.1. Panel Threshold Model

The threshold regression developed by Hansen and Gonzalez et al. uses the relevant sample data reflecting causal variables to estimate threshold values and the significance of parameters through the model of divided sample groups [46]. When a certain economic parameter reaches a certain critical value, the direction or quantity of another economic parameter will undergo a structural mutation, and the critical value of this economic parameter is the threshold value [47]. It was presented by Tong as a viable econometric method which has become quite popular in academic research [48]. The advantage of this model is that it avoids the randomness of traditional analysis and has no fixed nonlinear equation form. Second, the number of samples determines the number of threshold values endogenously, and the threshold is estimated with full consideration of the characteristics of data samples. The model is described as follows:

(8) $$\begin{array}{c} Y_{it}=\mu_{i}+\vartheta_{1}X_{it}I\left(q_{it}\leq \gamma_{1}\right)+\vartheta_{2}X_{it}I\left(\gamma_{1}<q_{it}\leq \gamma_{2}\right)+\vartheta_{3}X_{it}I\left(q_{it}>\gamma_{2}\right)\\ +\overset{n}{\underset{j=1}{\sum }}Control_{ijt}+\varepsilon_{it} \end{array}$$

where $Y_{it}$ represents the explained variable, $X_{it}$ represents a vector of variables, $q_{it}$ is threshold variable, $\gamma_{1}$ and $\gamma_{2}$ are threshold values, and $\sum_{j=1}^{n}Control_{ijt}$ represents the control variables. Equation (3) is a multi-threshold model. I( ) is an indicator function of 0 or 1, subscript i indexes the individual, and the subscript t indexes the time. The slope coefficients $\gamma_{1}$ and $\gamma_{2}$ represent the influence of variable $X_{it}$ on explained variable $Y_{it}$. If the estimation of intermediate $r_{1}<q_{it}\leq r_{2}$ is reduced, then it will become a single-threshold model.

In this paper, to investigate the relationship between eco-efficiency and ERs, the corrected efficiency value through bootstrap method is selected as the dependent variable, and the ER is the core explanatory variable. Ownership structure (OS) and the degree of COM are chosen as control variables. Thus, RD, IS, and LM are selected as threshold variables. The threshold regression model is as follows:

(9) $$\begin{array}{c} \text{Eco-efficiency}=\mu_{i}+\vartheta_{1}ER_{it}I\left(q_{it}\leq \gamma_{1}\right)+\vartheta_{2}ER_{it}I\left(\gamma_{1}<q_{it}\leq \gamma_{2}\right)\\ +\vartheta_{3}ER_{it}I\left(q_{it}>\gamma_{2}\right)+\tau_{1}OS_{it}+\tau_{2}COM_{it}+\varepsilon_{it} \end{array}$$

where eco-efficiency denotes the values of eco-efficiency corrected by bootstrap method, $ER_{it}$ denotes ER; $OS_{it}$ means OS; $COM_{it}$ means the level of COM; $q_{it}$ is threshold variable. The coefficient and variable matrices are δ and Q, respectively:

$$\delta =\left(\begin{array}{c} \tau_{1}{}_{1}\\ \tau_{2}\\ \vartheta_{1}\\ \vartheta_{2}\\ \vartheta_{3} \end{array}\right)\;Q=\left(\begin{array}{c} OS_{it}\\ COM_{it}\\ ER_{it}I\left(q_{it}\leq \gamma_{1}\right)\\ ER_{it}I\left(\gamma_{1}<q_{it}\leq \gamma_{2}\right)\\ ER_{it}I\left(q_{it}>\gamma_{2}\right) \end{array}\right)$$

$$\text{Eco-efficiency}=\delta^{T}Q_{it}\left(\gamma \right)+\mu_{i}+\varepsilon_{it}$$

The above function in matrix form is:

$$Y^{*}=\delta Q{\left(\gamma \right)}^{*T}+e^{*}$$

For any given threshold value γ, coefficient δ can be calculated as:

$$\delta_{\gamma }={\left(Q^{*}{\left(\gamma \right)}^{T}Q^{*}\left(\gamma \right)\right)}^{-1}Q^{*}{\left(\gamma \right)}^{T}Y^{*}$$

The value of residual can be easily estimated as:

$$e^{*}Y=Y^{*}-Q^{*}\left(\gamma \right)\delta_{\gamma }{}^{T}$$

The sum of squared errors can be calculated as:

$$SSE\left(\gamma \right)=e^{*}\gamma^{T}e^{*}\left(\gamma \right)=Y^{*}{}^{T}\left[I-Q^{*}{\left(\gamma \right)}^{T}{\left(Q^{*}{\left(\gamma \right)}^{T}Q^{*}\left(\gamma \right)\right)}^{-1}Q^{*}\gamma^{T}\right]Y^{*}$$

where the threshold value can be estimated as follows:

ϵ^=mϵarginSSE1(ϵ)

The estimated scheme search can be applied for single, double and triple–threshold models.

#### 3.2.2. Indicator Description and Data Processing

(1). Dependent and Independent Variables

The dependent variable is eco-efficiency, which is measured by the values corrected by bootstrap—DEA model. According to the previous studies, we followed the practice of Ederington et al., and Levinson and Taylor to select the cost of emission reduction or investment in pollution control to indicate the severity of ER [49,50]. In this paper, ER is measured by the intensity of investment in pollution control. The higher the value is, the stronger the government’s ER will be.

(2). Control Variables

According to the existing literature [25,26,27,28,29,30,31,32,33,49], the eco-efficiency of land use is affected by many factors in addition to the ER. (1) The freedom of market economy indicates the process of marketization, which can improve the efficiency of economic factor allocation through market mechanism. The OS is measured by the proportion of the number of state-owned enterprises and total employment. (2) Local governments take advantage of the one-sided pursuit of economic growth and fiscal revenue to win political promotion opportunities, neglecting not only the environmental governance and investment, but also the important institutional defect [28]. Referring to the practice of Wang and Xu, the number of annual changes of party and government leaders in each province was used as an intermediate variable to indicate the degree of COM [50]. If there is a change of party and government heads in the province in that year, the intermediate variable of the province is assigned as the number of changes of party and government heads in other provinces minus the number of changes of party and government heads in the province. Given no replacement of the provincial party and government heads in that year, the intermediate variable of the province is the number of replacement of other party and government heads in that year. The replacement times of the provinces are then standardized as follows:

(10) $$com_{m,n}=\frac{\sum_{m\neq n}^{P_{n}}k_{m,n}}{2P_{n}}$$

where, $P_{n}$ represents the number of provinces and districts in the $n_{th}$ year, and b represents the number of the replacement of party and government heads (secretaries and deputy secretaries, governors and deputy governors) in the $n_{th}$ province.

(3). Threshold Variables

The secondary industry accounts for a relatively large accompanied with large amounts of energy consumption, and the proportion of industrial added value accounts in regional GDP is selected to IS. The proxy variable to measure RD is defined as the regional investment of research and development. LM is represented by the ratio of the number of land lots to the total number of transferred land lots in the primary land market. The method of land transfer in China has changed from the negotiated transfer to the “bidding, auction and listing” transfer, avoiding the government behavior of “free riding.” Through rational regulation by the market, the transaction of land use right is no longer controlled by the government monopoly. In the transaction, the party with the highest bid gets the land use right. Good market mechanism effectively regulates and controls land resources and greatly improves the efficiency of resource utilization.

## 4. Results and Analysis

### 4.1. Analysis of Eco-Efficiency Results

Bootstrap-DEA model was used to calculate the average eco-efficiency of 30 Chinese provinces from 2004 to 2016, and then compared with the uncorrected efficiency results (Table 2). The comparison results show that the average efficiency value of each year after deviation correction was lower than that of the traditional efficiency measurement method, and all average errors were above 0, which indicates that the eco-efficiency value directly measured by the fixed directional distance function was higher than the real value. Traditional measurement methods were highly dependent on the original data, which could not show the characteristics of non-parametric statistics. Hence, the results modified by Bootstrap method were more reliable and authentic. In this paper, we chose to analyze the corrected value of eco-efficiency.

Table 2 shows that the overall eco-efficiency in China was not so efficient compared with the optimal frontier level and showed a trend of declining first and then rising. During 2004 to 2009, the eco-efficiency values in China were in a fluctuating growth trend. As the last year of China’s ‘Tenth Five Year Plan’, the government hoped to obtain the biggest economic boost to reflect their achievements in the shortest possible time due to China’s promotion mechanism. As a result, the government failed to consider ecological protection while pursuing economic development. Thus, the level of eco-efficiency declined in 2005. During 2008 to 2009, the level of eco-efficiency increased, which was consistent with the research results of Yang and Huang, proving that the fluctuation of eco-efficiency in China was synchronized with economic development [51,52]. Since the reform and opening up, the southern area (including Guangdong, Guangxi and Hainan) has achieved the highest level of eco-efficiency among the seven regions by encouraging international trade and investment, integrating technology, equipment, and management experience with international standards. Rapid economic development ensures that a region has the conditions to expand investment demand for high-tech industries and environmental governance, which not only promotes technological progress, but also improved the utilization of resources and reduces ecological pollution. In November 2008, the executive meeting of the state council of China put forward the fiscal and monetary policies to cope with the international economic crisis in 2008 and stabilize the economy, which became the “four trillion yuan investment plan”. One of the ten measures in the plan pointed out that key energy conservation and emission reduction projects should be supported and ecological and environmental protection should be strengthened. The reason for the improved efficiency levels seen in 2008, which continued into 2009, was the Beijing Olympic Games in 2008. In preparation for the 2008 Beijing Olympics, China had taken several pollution control measures to improve air quality. According to the State Key Laboratory of Environmental Chemistry and Ecotoxicology at the Chinese Academy of Science, the government took numerous measures to improve air quality during the 2008 Olympic Games. Pollution control measures included improvement of vehicle emission standards, IS adjustment, relocation and closure of heavily polluting industrial enterprises, adjustment of energy structure, and control of coal pollution [53]. From 2009 to 2010, the eco-efficiency decreased rapidly, making ecosystem suffer serious damage because of the international financial crisis in 2008. To cope with the crisis brought by the financial crisis, China proposed a four-trillion-yuan investment plan in 2009, which concentrated 80% of the investment in resource-intensive industries to boost domestic demand. While promoting economic recovery, it led to the overburdened capacity of resources, aggravation of pollution discharges and the decline of eco-efficiency [54]. A large gap of eco-efficiency was observed between 2011 and 2010, because China required coal consumption to be 63% and 58% more important than that in the Eleventh Five-Year Plan of China (2006–2010) during the Twelfth Five-Year Plan (2011–2015) and the Thirteenth Five-Year Plan (2016–2020), respectively [55]. To meet the requirements of the plan, China started to strictly control energy consumption in 2011, greatly improving eco-efficiency in 2011 and maintaining its relatively stable level until 2016.The average efficiency value during period examined was only 0.6860. If eco-efficiency is at the production frontier, then the efficiency value would be 1. Therefore, the gap between production frontier efficiency and the average efficiency value in the region examined can be considered as the room for improvement of eco-efficiency, which is 31.4%. Eco-efficiency has not reached the ideal state overall, indicating that the regional economic development and the optimal allocation of resources in China still has the potential for improvement.

**Figure 1:**
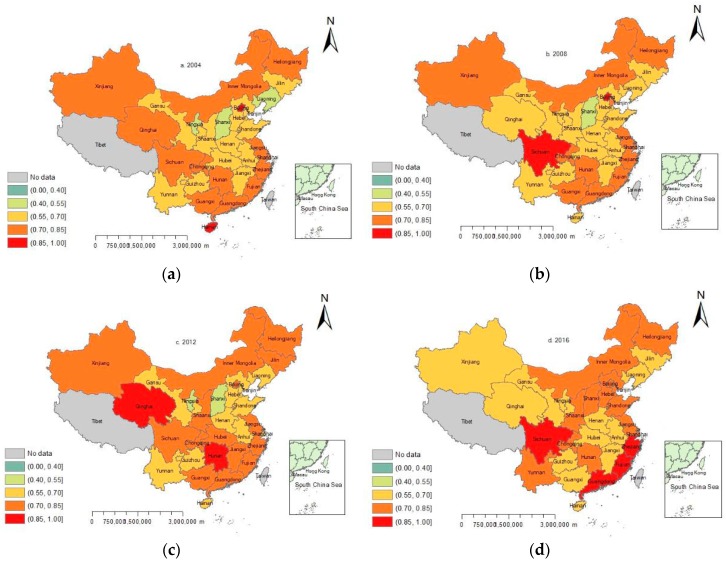
Spatial distribution of eco-efficiency of China in (**a**) 2004, (**b**) 2008, (**c**) 2012, and (**d**) 2016.

The spatial distribution pattern of China’s eco-efficiency in 2004, 2008, 2012 and 2016 are presented successively with an interval of four years. The levels of eco-efficiency values are divided into four grades: eco-efficiency ≤ 0.55 represents lower efficiency, 0.55 < eco-efficiency ≤ 0.7 represents medium efficiency, 0.7 < eco-efficiency ≤ 0.85 represents good efficiency, and 0.85 < eco-efficiency ≤ 1 represents higher efficiency. ArcGIS 10.0 software (ESRI, Redlands, CA, USA) was used to draw the spatial and temporal distribution patterns of eco-efficiency in China (Figure 1).

**Figure 2:**
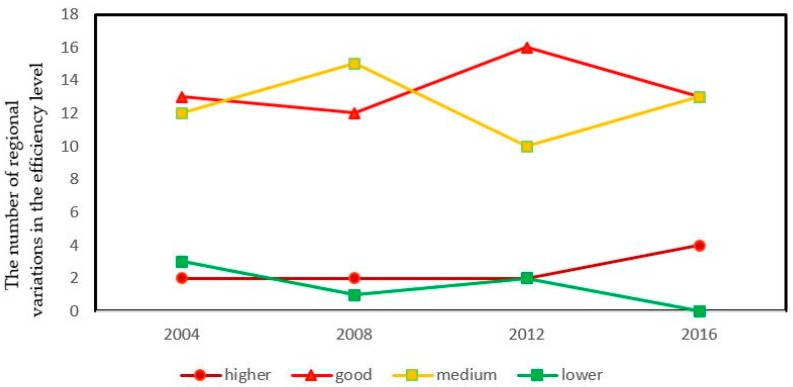
Temporal trend of eco-efficiency of China in 2004, 2008, 2012, and 2016.

Overall (Figure 1), the spatial distribution of eco-efficiency in China was of inter-provincial difference and non-equilibrium in 2004, 2008, 2012 and 2016. According to Figure 2, The proportion of regions with higher eco-efficiency in China was flat and then increased, the portion of regions with better eco-efficiency increased first and then decreased, the proportion regions with medium efficiency presented a change trend of increase–decrease–increase and the proportion of regions with lower efficiency changed from decreasing to increasing and became 0 finally. 

From the perspective of region, the regions with higher eco-efficiency in China were only Beijing and Hainan in 2004; the regions with good efficiency were distributed along the longitudinal axis from the southeast costal area to the central area and northeast area to the northwest area; the regions with medium efficiency were centered on Henan and related to the east, west, and south. In 2008, the areas with higher efficiency changed from south to southwest, the efficiency of Qinghai worsened, while the efficiency of Sichuan increased and the quantity of lower efficiency areas decreased.

In 2012, the regions with higher efficiency were transformed from Beijing and Sichuan into Qinghai and Hunan, the efficiency level of areas on both sides of the vertical axis of Hubei-Jiangxi was improved compared with that of 2008, whereas the number of areas with lower efficiency was increased. In 2016, the number of regions with higher efficiency in land use in China doubled, most of which were concentrated in the southeast coastal area, whereas the number of regions with good efficiency decreased and the level of efficiency in northwest was reduced. The overall eco-efficiency in China generally improved.

**Figure 3:**
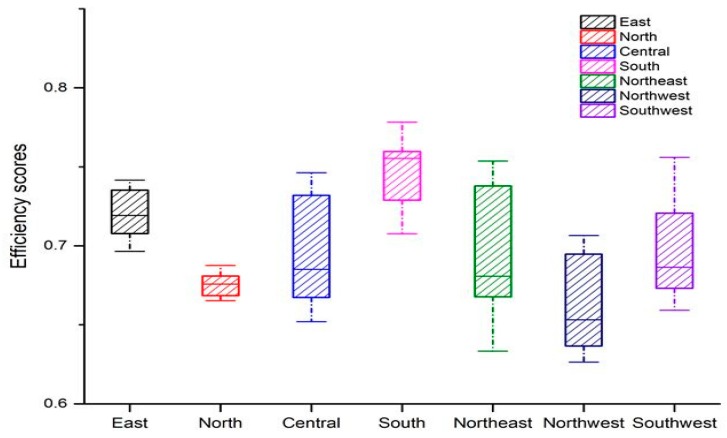
Changing trends of eco-efficiency in China’s seven regions from 2004 to 2016. East: Shanghai, Jiangsu, Zhejiang, Anhui, Fujian, Jiangxi, and Shandong; North: Beijing, Tianjin, Shanxi, Hebei, and Inner Mongolia; Central: Henan, Hubei, and Hunan; South: Guangdong, Guangxi, and Hainan; Northeast: Heilongjiang, Jilin, and Liaoning; Northwest: Shaanxi, Gansu, Qinghai, Ningxia, and Xinjiang; Southwest: Chongqing, Sichuan, Guizhou, and Yunnan.

Figure 3 depicts the eco-efficiency on a map of China’s seven regions. The entire China performed fairly well on the production frontier, which indicates that economic growth in China decoupled well from resource inputs and environment pressure. The values of seven regions’ eco-efficiency were all over 0.6, whereas northwest area performed worst among them with the average values less than 0.7, implying more than 30% of the resource inputs was not converted into GDP. South area performed best among them with the average values more than 0.75, implying less than 25% of the resource inputs was wasted in these regions as indicated by the red color in Figure 1.

### 4.2. Testing of Threshold Effects and the Analysis of Threshold Regression

The testing process of panel threshold regression includes determining whether the threshold effect exists. If it exists, then the threshold value and the significance level of the threshold effect will be further estimated. On the basis of the above analysis, the panel threshold model was processed with Stata MP 14.0 software in this paper. One thousand bootstrap replications were used to test whether the model has threshold effects and estimate the threshold value of each variable (Table 3).

As shown in Table 4, the panel threshold effects were tested by considering three threshold variables (RD, IS, and LM) on eco-efficiency under the constraint of land use carbon emission in an attempt to determine the non-linear connection between ERs and eco-efficiency.

(1) When RD was taken as the threshold variable, ERs had a double-threshold effect on eco-efficiency. A double-threshold model was used for regression analysis, and the influence direction and degree were affected by the value of RD. According to Table 4 and Table 5, the values of threshold were 0.3735 and 0.4175 for eco-efficiency. As RD exceeded the threshold point, the level of carbon-reduction technology increased and then exerted a better influence on eco-efficiency. The threshold points were identified at 0.3735 and 0.4175 with coefficients at −0.0337, −6.4322 and 0.8347 respectively, which means that the effect of innovation on eco-efficiency remains negative until the level of technical innovation exceeded the second threshold. With an increase in RD level, the effort of ER on eco-efficiency followed a U-shaped curve. As the RD reached a higher level, the effect of the coefficient of ER on eco-efficiency became positive gradually. The implementation of ER could promote the eco-efficiency, which is similar to the finding of Luo and Wang [56]. The improvement of RD could boost the innovation of clean production technology, which could accomplish clean production at the source, reduce the intensity of resource consumption, and obtain more expected output while reducing more undesirable output. The pollutant emission will not be reduced in the short term while the technical innovation level was low. By contrast, the ERs will curb the pollutant and promote eco-efficiency. The eco-efficiency of environmental regulations under the constraint of carbon emission conforms to the Porter hypothesis. Reasonable ERs can reduce the cost by stimulating the innovation of green production technology to achieve a win-win situation between the environment and outputs. Through the analysis of the sample data, only nine provinces including Beijing, Shanghai, Tianjin, Hubei, Sichuan, Shaanxi, Gansu, Jilin and Liaoning crossed the second threshold of RD in 2004, and the rest did not exceed the first threshold. By 2010, only Hainan did not cross the first threshold, whereas all other provinces exceeded the second. This phenomenon showed the importance of scientific and technological innovation to the development of land gradually coming into focus. In the initial stage of the study, most of the nine provinces that exceeded the second threshold were economically developed regions or regions with great development potential. Compared with 21 other provinces, the science and technology resources of these regions had more advantages and higher investment in education finance. The country’s increasing emphasis on science, technology and education also contributed to the gradual improvement of regional eco-efficiency.

(2) The regression parameters related to LM are given in Table 4 and Table 5. As expected, ERs had significant influence on eco-efficiency, which had a double threshold, and the influence direction and degree were affected by the value of LM. The influences of ER on eco-efficiency differed at various LM levels. When the degree of LM exceeded 0.175, and was lower than 0.3219, the coefficient of ER on eco-efficiency changed from 4.1022 to −0.4304. The coefficient was 1.2621 when the LM was more than 0.3219. This indicated that the influence of LM on regional eco-efficiency was related to its threshold values, and when land marketization crossed the threshold values, the direction and degree of influence on regional eco-efficiency changed, and a difference was observed in the degree of positive promotion of eco-efficiency before and after the LM level crossed the threshold level. In 2004, five provinces, namely, Beijing, Shanghai, Zhejiang, Sichuan, and Xinjiang, did not cross the first threshold, whereas 11 provinces, namely, Shanxi, Heilongjiang, Shannxi, Hainan, Jiangxi, Guizhou, Henan, Hunan, Yunnan, Qinghai, and Gansu, did exceed the second threshold. By 2010, all provinces had crossed the first threshold, with only four provinces between the first and the second threshold of LM by the sample observation, which were Guangxi, Guangdong, Hunan, and Yunnan. With an increase in LM level, the effort of ER on eco-efficiency followed a U-shaped curve because its level could adjust the structure of supply and demand of land market. The “bidding, auction and listing” supply mode increased the land remising price, and LM increased the acquisition cost of construction land and land fiscal revenue, prompting local governments to increase the supply of construction land. LM brings about the increase of monetary liquidity, and the reduction of land and capital factor price leads to the decrease of industrial land price. Due to the uniqueness of China’s land use system, the government can supplement the price of industrial land by obtaining more land income from commercial and residential land, and the labor cost of enterprises is increasing. Therefore, land finance will promote the development of the secondary industry in the short term, and the large consumption of resources will lead to the increase of pollution emissions, thereby negatively affecting the ecological efficiency. However, in the long run, the peak value of the secondary industry would be brought forward and deindustrialization effect will occur. A positive effect on eco-efficiency is observed in the later stage.

(3) When IS was as a threshold variable to study the role of ERs on eco-efficiency, a single threshold effect was observed. The influence direction and degree were affected by the value of IS level. According to Table 4 and Table 5, the threshold value was 0.25 for eco-efficiency. When the IS was below the threshold value of 0.25, the estimated coefficient was −1.4679. In this stage, the increase of the IS level led to the decline of eco-efficiency. When the IS level crossed the threshold value of 0.25, the estimated coefficient was 1.0899. During the study period, only in 2007 and 2008 did all provinces cross the threshold of IS. In other years, only Hainan and Beijing did not cross the threshold successively. The acceleration of the IS level contributed to the improvement of the eco-efficiency level. The single threshold divided the samples into two sections, and different levels of IS had different effects on eco-efficiency.

Two reasons explained the positive and negative effects of LM on eco-efficiency. First, the market-oriented reform of land transfer increased the competition in the land transaction market, and the housing price was driven upward [57], which intensified the dependence on land transfer and real estate and would inevitably cause the consumption of energy and building materials and aggravate the environmental pollution [14]. This further explained the phenomenon that the coefficient was negative when the LM failed to break through the threshold value. Second, with the long-term implementation of LM reform, the reform of system of “bonus” to optimize the configuration of land resources improved resource use efficiency [58] as well as reduced the waste of resources and played a significant role in the improvement of the eco-efficiency, explaining why LM crossed the threshold value and the positive direction of the coefficient value. Furthermore, the government was more inclined to measure the performance of the officials by the level of green economy. To improve the official performance, the governments proposed more appropriate ERs for the reduction of pollution emissions to improve the level of eco-efficiency and achieve the goal of Green Cyclical Economy.

According to Table 5, OS had a positive effect on regional eco-efficiency. Although state-owned enterprises have inherent defects in the arrangement of property rights, they are faced with “survival dilemma” and “growth drag” in the market environment [59]. However, with the implementation of the property right reform, the state-owned enterprises preliminary established a modern corporate governance structure with clear property right and responsibility, as well as separate government functions and scientific management, which have enhanced competitiveness and vitality of the enterprises. The survival dilemma has been greatly alleviated, and the technological progress and production efficiency of state-owned enterprises have been greatly improved [60]. The total factor productivity of state-owned enterprises after property right reform has exceeded that of non-state-owned enterprises in recent years. The improvement of resource utilization rate increases the economic benefits of enterprises, which is helpful to the improvement of eco-efficiency. The degree of COM had a negative effect on regional eco-efficiency. Local officials are still dominated by large-scale and extensive investment during their tenure, and have not paid sufficient attention to energy conservation and emission reduction. Not much efforts were exerted to reduce the carbon emission in the year before or in the year before the key party congress promotion. However, after the party congress, that incentive waned. In the long run, no normal, endogenous mechanism has been formed, which still has a negative effect on the eco-efficiency of the regions.

## 5. Conclusions and Policy Implications

Using panel data for 30 provinces in China from 2004 to 2016, the eco-efficiency under the constraint of land use carbon emission of 30 provinces in China was measured, and the nonlinear relationship between ERs and eco-efficiency was investigated. At the same time, LM, IS, and RD were chosen as threshold variables. Panel threshold model analysis confirmed that the effects of ERs on eco-efficiency under the constraint of land use carbon emission were highly connected to the three different threshold variables affecting land use carbon emission:

(1) Based on land use carbon emissions as an undesired output, this paper measured the eco- efficiency of 30 provinces in China from 2004 to 2016 with bootstrap method. The results indicated that the average eco-efficiency value of each province after deviation correction was lower than that of the traditional eco-efficiency measurement method, and the eco-efficiency directly measured by the mixed directional distance function was higher than the real value. The spatial distribution of eco-efficiency in China was different and unbalanced among provinces, and the distribution of efficiency level had significant agglomeration effect. In terms of regions, the number of regions with high eco-efficiency in China increased gradually, and the distribution range from the northeast and northwest longitudinal lines was gradually concentrated in the southeast coastal area. The number of regions with high eco-efficiency decreased, and the efficiency level of all 30 provinces was higher than the lower level. The overall eco-efficiency of China improved.

(2) During the sample period, under the influence of different levels of RD, IS, and LM, the change of the regional eco-efficiency would be different and presented significant threshold effects. Hence, while improving regional eco-efficiency, we should not only pursue the output and the speed of economic development, but also pay attention to the improvement of ecological environment as well as the ER within a reasonable range. Compared with RD and IS, ER had a more noticeable effect on eco-efficiency under the constraint of land use carbon emission when the level of LM was used as the threshold variable. Therefore, in the process of developing Green Circular Economy, the government should adopt more appropriate land transfer policies according to local conditions to improve eco-efficiency.

(3) The nonlinear relationship between the IS and eco-efficiency presented insightful suggestions for promoting eco-efficiency. Through the “structural effect,” the IS forced the city to improve the technological level and the efficiency of resource allocation to reduce pollution emissions and optimize eco-efficiency. In China, the IS level exerted a considerable positive effect on eco-efficiency only when it was maintained at a suitable level. Otherwise, IS decreases the eco-efficiency level. The promotion of officials depends on the election of the party congress. This intensive assessment of promotion idea does not really consider green achievements, such as energy conservation, and emission reduction in the promotion standard. The old promotion idea should be abandoned, the proportion of energy conservation and emission reduction assessment should be increased, and the assessment should be normalized.

(4) To improve the regional eco-efficiency of China and promote sustainable development, the following suggestions are put forward: First, strengthen the financial investment in science and technology, create a good environment for technology research and development, realize green and clean production, drive pollution reduction and improve the level of eco-efficiency. Second, grab from system root to increase the flexibility of election time for promotion of officials, and the proportion of performance evaluation on energy conservation and emission reduction. Third, pollution emission remains an important factor restricting eco-efficiency. While controlling pollution emission, the formulation and implementation of ERs should be combined with the regional status quo to guide the broad participation of the public and mobilize the initiative of pollution subjects to implement emission reduction.

Although this study estimated the threshold effect of ERs on eco-efficiency under the constraint of land use carbon emission based on bootstrap model and panel threshold model, some limitations remain. The eco-efficiency presents certain agglomeration characteristics in space, so the next step of the study can be combined with the spatial econometric model for a more in-depth analysis.

## Figures and Tables

**Table 1:** Summary of input and output indicators.

Index	Parameters	
Input	Land average capital stock	
	Land average labor	
	Land average energy consumption	
Output	Desirable output	GDP
	Undesirable output	CO_2_

Note: GDP is an abbreviation of Gross Domestic Product.

**Table 2:** Comparison between the eco-efficiency values and their modifications.

Year	Eco-Efficiency	Eco-Efficiency after Modification	Bias	Derivation	Confidence Intervals
2004	0.7872	0.6979	0.0893	0.0488	[0.6052, 0.7740]
2005	0.7740	0.6776	0.0964	0.0523	[0.5784, 0.7595]
2006	0.7750	0.6803	0.0946	0.05198	[0.5822, 0.7618]
2007	0.7720	0.6764	0.0955	0.0510	[0.5790, 0.7570]
2008	0.7813	0.6870	0.0943	0.0499	[0.5880, 0.7661]
2009	0.7904	0.6991	0.0913	0.0487	[0.6028, 0.7764]
2010	0.5871	0.4756	0.1114	0.0553	[0.3777, 0.5687]
2011	0.8085	0.7178	0.0906	0.0490	[0.6213, 0.7931]
2012	0.8132	0.7253	0.0878	0.0488	[0.6280, 0.7990]
2013	0.8116	0.7238	0.0878	0.0491	[0.6232, 0.7965]
2014	0.8117	0.7238	0.0880	0.0488	[0.6272, 0.7972]
2015	0.8049	0.7117	0.0933	0.0505	[0.6152, 0.7897]
2016	0.8131	0.7244	0.0886	0.0464	[0.6349, 0.7962]

**Table 3:** Summary statistical for variables.

Variables	Sum	Minimum	Maximum	Mean	Standard Error
Eco-efficiency	390	0.0025	0.9086	0.6861	0.1433
ER	390	0.008	0.1857	0.0424	0.0284
OS	390	0.0168	0.8343	0.1431	0.1308
RD	390	0.0491	6.6651	1.7399	2.4396
IS	390	0.197	48.9	3.6374	11.163
COM	390	0.7333	2.9167	1.5089	0.5941
LM	390	0.0429	5.9249	0.6065	0.3911

Note: ER, OS, RD, IS, COM and LM are abbreviation of environmental regulation, ownership structure, research and development, industrial structure, official competition and land marketization, respectively.

**Table 4:** Test on threshold effects and threshold value estimation.

Thresholds Variables	Number of Thresholds	F-Statistic	Threshold Value	95% Confidence Interval
**Technical Innovation (RD)**	Single	12.41 ***	0.46	[0.42, 0.47]
	Double	17.05 **	0.3735	[0.3506, 0.3900]
			0.4175	[0.4050, 0.4235]
**Industrial Structure (IS)**	Single	8.55 **	0.25	[0.240, 0.257]
**Land Marketization (LM)**	Single	7.52 **	0.1750	[0.1355,0.1797]
	Double	9.78 **	0.1750	[0.1437, 0.1797]
			0.3219	[0.2638, 0.3221]

Note: (1) *p* value and threshold value were obtained by Bootstrap 1000 times; (2) *** *p* < 0.01, ** *p* < 0.05, * *p* < 0.1.

**Table 5:** Estimation results of panel threshold model parameters.

Parameter	Coefficient	Parameter	Coefficient	Parameter	Coefficient
OS	0.4375 ** (0.1811)	OS	0.4855 *** (0.1848)	OS	0.6790 *** (0.193)
COM	−0.0776 *** (0.0105)	COM	−0.0882 *** (0.1064)	COM	−0.0834 *** (0.011)
$\mathrm{ER}\left(rd\leq \gamma_{1}\right)$	−0.337 * (0.2057)	$\mathrm{ER}\left(lm\leq \gamma_{1}\right)$	4.1022 *** (0.0863)	$\mathrm{ER}\left(is\leq \gamma_{1}\right)$	−1.4679 ** (0.0534)
$\mathrm{ER}(\gamma_{1}<rd\leq \gamma_{2})$	−6.4322 *** (0.0767)	$\mathrm{ER}(\gamma_{1}<lm\leq \gamma_{2})$	−0.4304 * (0.4378)		
$\mathrm{ER}(rd>\gamma_{2})$	0.8347 ** (0.1336)	$\mathrm{ER}(lm>\gamma_{2})$	1.2621 *** (0.0314)	$\mathrm{ER}(is>\gamma_{1})$	1.0899 *** (0.0303)
R^2^	0.1882	R^2^	0.1423	R^2^	0.1322

Note: (1) the standard deviation of each coefficient was shown in brackets. (2) *** *p* < 0.01, ** *p* < 0.05, * *p* < 0.1.
